# Decoding tumor–stroma interactions: from molecular crosstalk to therapeutic targets

**DOI:** 10.1002/2211-5463.70108

**Published:** 2025-10-01

**Authors:** Isabel Fabregat

**Affiliations:** ^1^ TGF‐β and Cancer Group, Bellvitge Biomedical Research Institute (IDIBELL) and CIBEREHD L'Hospitalet de Llobregat Barcelona Spain

## Abstract

With this “In the Limelight: Tumor‐Stroma Interactions” special issue, *FEBS Open Bio* aims to highlight the relevance of the tumor stroma cells, and their interactions with the tumor cells, in the progression of cancer.

**Abbreviations**
apCAFs: antigen‐presenting CAFs
CAFs: cancer‐associated fibroblasts
ECM: extracellular matrix
iCAFs: inflammatory CAFs
MAFs: metastatic‐associated fibroblasts
MyCAFs: myofibroblast‐like CAFs
NSCLC: non small cell lung cancer
TME: tumor microenvironment

Cancer cells are embedded within a diverse array of nonmalignant cell types, all collectively surrounded by the extracellular matrix, a dynamic, intricate three‐dimensional network of biomolecules with both structural and functional roles. Understanding the multifaceted functions of the matrix is crucial for advancing fields such as cancer research, given its integral and dynamic role in the tumor microenvironment. In this special issue, Skandalis and cols. [1] address the complexity of this amalgam of structures and functions, emphasizing the pivotal role of the tumor microenvironment (TME) in tumor maintenance and progression. They describe several bioactive macromolecules within the matrix, such as proteoglycans, hyaluronan, collagens, elastin, and matricellular proteins, as well as their cellular receptors, including integrins and CD44. These bioactive components can exhibit either tumor‐suppressive or tumor‐promoting properties. Moreover, some macromolecules, depending on their form, structure, or conformation, may exert opposing effects. Examples include hyaluronan (high molecular weight vs. low molecular weight) and perlecan (intact proteoglycan vs. C‐terminal endorepellin fragment). This insightful article underscores the matrix as the tumor cell's ultimate “tango partner”, facilitating growth, expansion, and survival. The work aims to pave the way for novel matrix‐based therapeutic strategies and the identification of potential biomarkers for tumor diagnosis and monitoring.

Tissue engineering of 3D *in vitro* models of the tumor microenvironment (TME) offers a valuable way to study how different components interact and influence cancer progression. There is a growing need for patient‐specific models that can closely mimic the complexity of tumors at different stages of the disease. In this special issue, Rafik et al. [2] review recent advances in 3D *in vitro* models of the TME, focusing on how they can be used to study the interactions between various elements of the tumor environment. They also describe the tools available to analyze these models. These improved 3D models allow researchers to study cancer in more realistic conditions and better understand the role of both cells and the surrounding matrix. However, 3D models still have some limitations that may affect their use in clinical applications. These include difficulties in fully reproducing the diversity of cancer cells, differences between the model and the human body, challenges in designing the 3D structure (such as which cells and materials to use), and problems with measuring and standardizing the models. This noteworthy article suggests that, in the future, machine learning could be used to predict the behavior of 3D models based on their composition, potentially accelerating the development of personalized cancer models. This would involve carefully selecting the right cells and materials, and closely controlling the conditions in which the models grow.

Beyond cell–cell interactions and biochemical signals, the tumor stroma is also defined by its unique mechanical properties, which are shaped by the composition and organization of the extracellular matrix (ECM). Cancer‐associated fibroblasts (CAFs) are the primary producers and remodelers of the stromal ECM, and the heterogeneity among CAF subtypes has recently become a major focus of research. In this special issue, de la Jara Ortiz *et al*. [3] discuss recent findings on the roles of different CAF subtypes and the development of 3D models to study tumor stroma mechanics *in vitro*. They describe quantitative techniques for measuring tissue mechanical properties across different scales and highlight both the similarities and differences among CAF subtypes. The authors also provide an overview of *in vitro* preclinical models designed to replicate the cellular and noncellular complexity of the TME. Understanding the molecular mechanisms that drive CAF plasticity is essential for advancing cancer biology, and more detailed studies on CAF origin and differentiation are needed. In this perceptive review, the authors highlight that the mechanical properties of the stroma, shaped by dynamic interactions among CAFs, the ECM, immune cells, and cancer cells, are increasingly recognized as key regulators of tumor growth and invasion, as well as promising diagnostic tools.

Notably, growing evidence indicates that cancer‐associated fibroblasts (CAFs) can exert either tumor‐promoting or tumor‐restraining effects. Current therapeutic strategies aim to shift this balance toward tumor‐restraining phenotypes. In this special issue, Bernardo *et al*. [4] review major advances in our understanding of tumor stromagenesis and CAF heterogeneity in both primary and metastatic tumors, with a particular focus on nonsmall cell lung cancer (NSCLC) and its frequent metastasis to the brain. Myofibroblast‐like CAFs (myCAFs) are predominant in solid tumors. However, studies have also identified inflammatory CAFs (iCAFs), characterized by high IL‐6 expression and other cytokines, and a smaller subset expressing antigen‐presenting genes (apCAFs). The authors note that, while consensus is emerging on key features of myCAFs and iCAFs, significant discrepancies and unresolved issues remain. Beyond facilitating metastatic colonization, CAFs may promote the expansion of metastasis‐initiating cells by inducing epithelial‐to‐mesenchymal transition (EMT) and stem‐like traits in cancer cells. However, the origin and tumor‐promoting roles of metastatic‐associated fibroblasts (MAFs) are still poorly understood. This noteworthy article also highlights growing interest in targeting CAF‐related mechanisms to overcome therapy resistance, particularly in NSCLC. In addition, the authors emphasize the critical role of smoking in epigenetically reprogramming the TGF‐β/SMAD3 pathway. These findings contribute to a clearer understanding of how CAF heterogeneity varies across tumor stages and histologic subtypes in NSCLC. Advances in this field may lead to new strategies aimed at eliminating or reprogramming tumor‐promoting CAFs and MAFs or enhancing tumor‐suppressive fibroblast populations. Such fibroblast‐targeted approaches hold great promise, especially when combined with chemotherapy and immunotherapy.

I would like to express my gratitude to all the authors for their inspiring contributions to this special “In the Limelight” issue of *FEBS Open Bio*.

## Conflict of interest

The author declares no conflict of interest.

## Author contributions

IF wrote the editorial.
